# Post-TB lung disease: pathogenesis, host-directed therapies, and the C3HeB/FeJ model for pre-clinical advances

**DOI:** 10.1128/iai.00523-24

**Published:** 2026-02-09

**Authors:** Boatema Ofori-Anyinam, Brian Nyiro, Padmini Salgame

**Affiliations:** 1 Department of Medicine, Center for Emerging Pathogens, Rutgers New Jersey Medical School 214907, Newark, New Jersey, USA; University of California Davis, Davis, California, USA

**Keywords:** tuberculosis, PTLD, C3HeB/FeJ, immunopathogenesis, host-directed therapy, post-TB lung disease

## Abstract

More than half of the people with microbiologically cured tuberculosis (TB) present with post-TB lung disease (PTLD). PTLD compromises long-term respiratory health and adds to the global burden of chronic lung diseases. Despite its prevalence, the mechanisms driving tissue damage in TB are not well understood. In this review, we discuss the global burden of PTLD, evaluate host-directed therapies as promising interventions, and highlight the C3HeB/FeJ mouse model as a powerful tool for advancing pre-clinical PTLD research.

## INTRODUCTION

Tuberculosis (TB) is still one of the leading infectious diseases worldwide, causing substantial illness and death. Each year, more than 10 million people develop active TB, and approximately 1.5 million lives are lost to the disease. While advances in diagnosis, treatment, and patient care have improved cure rates, they have also led to a growing population of TB survivors, estimated at 155 million globally in 2020 (1–3). Thus, an emerging public health concern is the high morbidity and mortality experienced by many individuals after achieving bacteriological cure. Although standard antimicrobial regimens are generally effective in eliminating *Mycobacterium tuberculosis* (Mtb), they rarely result in full restoration of lung health. Studies have consistently shown that over half of TB survivors exhibit lasting respiratory impairment, never regaining normal or baseline lung function, highlighting the heavy burden of post-TB lung disease (PTLD) (4, 5). Despite its prevalence, PTLD remains largely neglected in TB control strategies. In reviewing over 200 TB guidelines, only a few international documents referenced PTLD, and even those offered minimal guidance on diagnosis or management. The World Health Organization’s End TB Strategy itself makes no mention of PTLD, and most TB registries focus solely on outcomes during treatment, overlooking long-term disability (6, 7).

The consequences of PTLD are severe. TB survivors face mortality rates three times higher than those without prior TB and collectively lose an estimated 58 million of the 122 million disability-adjusted life years (DALYs) attributed to TB disease (6, 8, 9). These figures underscore the urgent need for systematic recognition and management of PTLD, as many patients never regain normal health or quality of life following treatment.

Using the Delphi technique at the First International Symposium for Post-TB lung health, an international expert panel defined PTLD as “evidence of chronic respiratory abnormality, with or without symptoms, attributable at least in part to previous tuberculosis.” (10). The condition encompasses a spectrum of problems, including chronic lung impairment (fibrosis, bronchiectasis, and chronic obstructive pulmonary disease [COPD]), abnormal spirometry patterns, airway stenosis, fibrosing mediastinitis, fibrothorax, and bronchopleural fistulae (6, 7, 11, 12).

PTLD develops due to the complex interplay between the host and the pathogen, co-morbidities, and the environment; thus, the pathological features of PTLD are varied. Therefore, there is an urgent need to deepen our understanding of what drives tissue damage in TB and explore the potential of therapies to prevent such damage. Current TB treatment regimens are not designed to address the ongoing inflammation and lung damage that accompany infection. This gap has prompted growing interest in host-directed therapies (HDTs), which aim to modulate the immune response, prevent irreversible tissue injury or destruction, and improve long-term outcomes. Unlike pathogen-targeted drugs, HDTs have the potential to reduce post-treatment disability while also mitigating risks of drug resistance and treatment failure. Therefore, expanding the drug discovery landscape to include HDTs is essential to overcome limitations associated with pathogen-targeted treatments, including drug resistance, tolerance, and the persistent or progressive tissue destruction that occurs even after bacterial clearance. Beyond reducing therapy duration, a key objective of incorporating adjunctive HDTs is to prevent irreversible lung damage caused by unbeneficial ongoing inflammation, thereby enhancing the long-term quality of life for TB survivors (13–20). Thus, it is important to study the effect of HDTs in appropriate models that accurately recapitulate the key features of TB pathology and host immune responses seen in humans, as such models would be essential for reliably assessing the efficacy, safety, and translational potential of HDTs. This would mitigate issues associated with generating results that may be misleading or inaccurately predict therapeutic outcomes in humans.

Therefore, the objectives of this review are to examine the global burden of PTLD, discuss HDTs being explored to reduce the burden, and highlight the utility of the C3HeB/FeJ mouse model as a preclinical tool to develop HDTs for PTLD.

## THE BURDEN OF PTLD

At least half of all TB survivors experience ongoing structural and functional lung damage after bacteriological cure, and those with residual lesions, such as cavitation, are at increased risk of developing recurrent disease, relapse, or progressive impairment. Many of the TB survivors go on to develop chronic airway obstruction, particularly COPD (8, 21–23).

Efforts to address the burden of PTLD are slowly emerging. For example, multicenter studies have evaluated pulmonary rehabilitation for patients with PTLD, a largely neglected intervention, and have sought to define predictors of PTLD by examining clinical, microbiological, immunological, and socioeconomic factors (9, 24–28). International consensus guidelines now recommend that every person who completes TB treatment must undergo post-treatment evaluation, including physical examination, a nutritional assessment, chest imaging, pulmonary function testing, a 6-min walk test (6MWT), and functional scoring (3, 10, 23, 29–31). Comorbid conditions such as HIV, diabetes, and cardiovascular disease should be recorded, and patients at risk for PTLD should be searched out and scheduled promptly for interventions such as airway clearance techniques and bronchodilator therapy (3, 6).

Large prospective studies, such as the TB Sequel project, are underway to primarily evaluate functional impairment and better characterize PTLD risk factors and outcomes, including the contribution of comorbidities (6, 28). DALYs provide a measure of the global disease burden and are estimated by combining the burden of mortality, measured as the years of life lost (YLL), and the burden of morbidity, measured as the years of life lived with a disability (YLD), due to a disease or condition. DALYs allow comparison between diseases that cause early death but minimal disability and those that result in disability without causing death. In the context of TB, DALYs help quantify the contribution of both post-TB mortality and morbidity to the overall burden of the disease. DALY-based analyses illustrate the scale of the problem, as current global TB burden estimates often assume full recovery at treatment completion, which significantly underestimates true disease impact. When post-TB mortality and morbidity are factored in, total TB DALYs increase by over 50%, with PTLD alone accounting for roughly half (3, 32).

People who survive TB face a higher risk of death and lose an average of 3.6 more potential years of life compared to the general population or matched controls without active TB, even after receiving adequate treatment. When post-TB mortality and morbidity were included, the estimated global TB burden increased by 6.1 million DALYs, which is a 54% rise. Considering additional TB-related conditions further raised this estimate to 20.1 million DALYs. These findings emphasize the importance of considering mortality and disability following treatment completion (post-TB sequelae) to fully capture the total impact of TB (3). Other studies have reported, similarly, that DALYs linked to PTLD constitute about half of the overall TB burden, and research indicates that YLL make up roughly a quarter of the total burden, while YLD account for approximately three-quarters (77%), mostly due to pulmonary impairment after TB (3, 33). Chronic pulmonary dysfunction after TB is responsible for the vast majority (97%) of YLD, with the remaining portion stemming from illness prior to treatment completion, including acute side effects. Ninety percent of projected lifetime YLD are concentrated among the most severely affected minority of survivors. The impact is disproportionately high in women, younger patients, HIV-positive individuals, and populations in high-incidence settings (32, 34). The interaction between TB and HIV, and the potential impact on PTLD, has been well discussed (32, 34–36). Interestingly, women were found to be more susceptible to lung disease, overall, driven by a combination of biological and social factors (37). However, despite global TB rates being consistently higher in men than in women (38, 39), why women suffer more from PTLD has not received sufficient attention.

Biologically, women have smaller lungs and airways, different immune responses, and hormonal influences that could heighten vulnerability to infections and inflammatory lung diseases (37). Furthermore, social disparities such as increased exposure to indoor air pollution from cooking fuels, limited access to healthcare, and gender-related delays in diagnosis could worsen outcomes in women (37). Women were more likely to have COPD and twice as likely to have chronic bronchitis, in contrast to the emphysema in men, after biological and social factors were adjusted (37). Hormones, like estrogen, have also been implicated in playing a role in hypersusceptibility of women to lung disease (37, 40). In fact, it was proposed that women generate lower amounts of reactive oxygen species (ROS), which protect them from some age-related biological processes, by having longer telomeres than men and a slower rate of telomere shortening, which, when accelerated, could cause cellular senescence (40–42). Since estrogen reportedly triggers telomerase activity, influencing the process by which telomere repeats are added to the ends of chromosomes, the susceptibility of women to lung diseases was proposed to be influenced by varying levels of estrogen as well (42).

These findings collectively highlight a substantial and underappreciated public health issue. Addressing PTLD will require integrated, long-term follow-up care, targeted rehabilitation, and proactive screening, in both men and women, without any disparities, for both physical and psychosocial sequelae.

## DIAGNOSIS OF PTLD: RECENT ADVANCES IN DETECTION AND EMERGING APPROACHES

Diagnosing PTLD is often complex because of its wide range of clinical presentations and its overlap with other chronic lung conditions. An integrated approach that combines clinical history, radiological evaluation, pulmonary function testing, and consideration of prior TB treatment would help provide a more reliable diagnosis (7).

PTLD resembles diseases such as COPD, other lung diseases, and bronchiectasis, and its symptoms, like persistent cough, shortness of breath, and repeated respiratory infections, are shared across these conditions, making clinical distinction challenging. Radiological findings further complicate diagnosis, as fibrotic and other changes typical of PTLD may appear similar to those seen in other lung pathologies (7, 43).

Pulmonary function tests are important in characterizing PTLD and help measure lung impairment, disease progression, and guide management strategies. Spirometry identifies airflow obstruction, while lung volume testing and diffusing capacity for carbon monoxide (DLCO) reveal restrictive patterns and gas exchange defects, respectively. Findings such as reduced forced expiratory volume in 1 second and lowered DLCO are often associated with PTLD and provide essential information on disease severity (7). A study from Malawi found that over 30% of participants had abnormal spirometry results by the end of treatment, and after 3 years, more than 80% of these individuals continued to exhibit abnormal pulmonary function test results, predominantly of obstructive nature (44–46). The clinical patterns of PTLD include diverse signs and symptoms, ranging from asymptomatic to severe disability. Clinical examinations must focus on respiratory rate, heart rate, and BMI on a routine basis (10).

During and after TB treatment, the tissue repair process in the lungs often results in structural alterations, including parenchymal tissue loss, which contributes to abnormal and obstructive patterns on spirometry (30). The mechanisms underlying airflow obstruction in TB are less well understood but are thought to be driven by bronchiectasis, bronchial stenosis, and immunological factors that can induce bronchial hyperresponsiveness, indicating that lung volume measurements should always complement spirometry to confirm abnormal patterns of pulmonary disease, since the type of ventilatory defect can be heterogeneous and vary in different populations (30). Notably, low DLCO can be found even in patients with normal spirometry and could be a more optimal option for lung function assessment in PTLD patients (25). The 6MWT helps evaluate patients with PTLD. This test is inexpensive to implement, simple, and very useful for studying functional limitations in TB survivors, as well as for designing appropriate rehabilitation programs for PTLD patients (9, 25, 47).

In individuals with significant impairments, assessment with arterial blood gas analysis or pulse oximetry can help detect hypoxemia. The criteria for initiating long-term oxygen therapy are consistent with those established for chronic airway diseases (30).

In terms of lung function assessment, cardiopulmonary exercise testing is considered the gold standard for assessing cardiorespiratory function due to its ability to depict how systems respond to physical exertion. However, it has been shown that the correlations between lung function patterns (assessed by either spirometry, DLCO, or plethysmography) and oxygen consumption measured during cardiopulmonary exercise testing are weak (30, 48).

Imaging is central to PTLD evaluation. Chest X-rays can reveal fibrotic opacities, cavitary lesions, and bronchiectasis, but may not be sensitive enough to detect subtle abnormalities. Recent advancements and progress in imaging, including computed tomography (CT) and positron emission tomography-computed tomography (PET-CT), are increasingly used in identifying PTLD (6). Computed tomography, particularly high-resolution CT, provides a more detailed assessment of the lung parenchyma, airways, pleura, and, indeed, the true nature of underlying lung damage after completion of TB treatment. It enables the precise visualization of fibrotic remodeling, bronchiectasis, and nodular lesions, making it a crucial tool for assessing the extent and severity of PTLD-related damage (6, 7).

Persistence of TB cavities is associated with poor treatment outcomes, and being able to identify the lesions that respond slowly can help optimize treatment regimens early and thus limit the development of PTLD. Unfortunately, there is no uniform, standardized, or universal scoring system for chest radiographs or CT scans that would comprehensively define PTLD, and this has been a hindrance to its adaptability. While access to CT imaging is unlikely to be widely available in the developing world, where the burden of TB is highest, its use within research settings can help phenotype PTLD (6).

Molecular imaging has gained prominence as a biomarker for various lung conditions. PET using 18F-fluorodeoxyglucose (18FDG) as a tracer to reflect glucose metabolism, in combination with CT imaging, constitutes a powerful imaging tool that provides valuable structural information, offering both structural and functional insights, and further enhances diagnostic accuracy (6, 49). A recent review analyzing CT scans from thousands of individuals revealed markedly higher detection rates of fibrosis and bronchiectasis, exceeding 80%, compared to chest X-rays. CT imaging also identified a broader spectrum of abnormalities, underscoring its value in diagnosing PTLD (50). Another study demonstrated that CT abnormality scores not only distinguished between patients with and without dyspnea but also correlated with the severity of dyspnea (51). Post-treatment PET-CT imaging revealed diverse outcomes: while some patients exhibited complete resolution of metabolic activity, others showed partial improvement, and a subset developed more intense or even new lesions. These new lesions may indicate differences in the progression of existing TB lesions or microevolution of bacterial subpopulations. Therefore, monitoring individual lesions, particularly their size and FDG uptake, can be critical for evaluating treatment response and anticipating PTLD risk (52). Nonetheless, the potential of imaging to serve as a powerful tool for predicting and confirming PTLD is compromised in asymptomatic patients or those who do not display lung function loss but may have abnormal imaging post-cure. Thus, while imaging is essential to evaluating PTLD characteristics, it cannot be used as a stand-alone to clinically confirm PTLD. Furthermore, repeated PET-CT imaging carries limitations, particularly regarding concerns over radiation exposure (6). Despite these challenges, PET-CT remains a valuable tool for characterizing PTLD stages, as differences in FDG uptake associate and align closely with lesion dynamics (53).

## ADDRESSING POST-PULMONARY TB SEQUELAE: THE BALANCE BETWEEN DAMAGE REVERSAL AND INJURY MITIGATION

Efforts to mitigate the long-term respiratory consequences of TB can be broadly divided into two complementary yet distinct strategies that span the entire disease continuum—prevention of injury versus reversal of established damage. Although these goals are interconnected, there is a clear distinction between them. Approaches to reverse post-TB lung damage seek to improve long-term function among survivors, whereas approaches to minimize lung injury during active TB attempt to prevent chronic sequelae from developing in the first place. Furthermore, both follow distinct lines of inquiry and rely on different biological mechanisms, evidence bases, and clinical endpoints. Preventive strategies depend on timely modulation of host responses during acute illness and on protecting the lungs from, or limiting, injury and excessive damage during active disease. In contrast, reversal strategies emphasize functional recovery and mitigation of chronic sequelae by focusing on how to restore or recover lung function once injury has already occurred. Addressing the full spectrum of TB-related morbidity will require therapeutic frameworks that integrate both preventive and restorative strategies, ensuring that survivors achieve not only cure but meaningful pulmonary recovery.

The first approach focuses on preventing or minimizing lung injury or tissue destruction during active disease through adjunctive or host-directed interventions, intended to modulate immune responses, reduce destructive inflammation, and preserve lung architecture before irreversible injury occurs. Interventions to minimize damage during active disease include the use of HDTs (e.g., doxycycline, metformin, and immunomodulators) that blunt destructive inflammation. These have shown promising biological signals but have mixed clinical outcomes, with important safety and translational questions still remaining (22, 54–58). The second seeks to reverse or rehabilitate established structural and functional impairments in individuals who have already completed treatment—an area increasingly informed by studies of pulmonary rehabilitation, airway-directed therapies, and emerging interest in antifibrotic strategies, with the aim of reversing established post-TB lung sequelae (8, 23, 59–61).

In this review, we focus on addressing preventive strategies using HDTs—emerging adjunctive therapies that aim to prevent or limit the inflammatory and proteolytic processes that drive tissue destruction during active infection.

## LUNG PATHOLOGICAL RESPONSE IN TB AS DRIVERS OF PTLD

PTLD arises from pathological lung remodeling driven by the interplay between Mtb and host immune responses. Both pro-inflammatory and anti-inflammatory processes can, depending on timing and tissue context or microenvironment, contribute to either protection or pathology (16, 62). Sustained inflammation during and after TB treatment, together with abnormal wound-healing processes, promotes continuous scar tissue formation, which progressively compromises lung function and increases vulnerability to respiratory complications. Furthermore, extensive tissue necrosis, combined with maladaptive repair processes, leads to irreversible injury and distortion of the lung structure (6, 7, 63, 64).

Across human and animal studies, multiple types of granulomas have been described, including non-necrotic, fibrotic, neutrophil-rich, calcified, caseous, and necrotic granulomas (65–68). Necrotic granulomas are characterized by the accumulation of lipid-laden foamy macrophages within the granuloma foci. Upon necrosis, these cells release their lipid contents into the granuloma core, driving caseous necrosis and predisposing to cavitation (69, 70). Matrix metalloproteinases (MMPs), particularly MMP-1 and MMP-9, have been strongly implicated in the degradation of the extracellular matrix (ECM) and in the progression from necrotic granulomas to cavitary lesions (71, 72). MMP-1 and MMP-9 are upregulated in TB and correlate with radiographic severity and tissue destruction. MMP-1 is a key driver of alveolar matrix breakdown in human TB (73–75). Neutrophil extracellular traps (NETs) further contribute to necrosis and caseation (70, 76). In nonhuman primates (NHPs), granulomas enriched for H3Cit, a NET-associated marker, were more likely to be necrotic than non-necrotic lesions (76). Necrotic and cavitary granulomas cause parenchymal destruction, bronchiectasis, and persistent airflow obstruction, core components of PTLD (77).

Neutrophil-rich granulomas can exacerbate immunopathology through neutrophil-driven tissue damage. Although neutrophils phagocytose bacilli, they are inefficient at killing Mtb (78, 79). During infection, the accumulation of neutrophils in the lungs promotes disease progression and immunopathological responses. Neutrophils are the largest category of infected cells in TB patients (80), and their depletion in chronic murine infection reduces lung pathology and bacterial burden (73). Mtb-induced necrosis of infected neutrophils fuels growth in macrophages (81), while the release of ROS, proteases, and NETs contributes to lung tissue destruction. Neutrophil-derived ROS, while contributing to bacterial control, also drive Mtb-induced necrosis, directly injure the epithelium, degrade the ECM, and promote the release of profibrotic mediators, reinforcing a cycle of irreversible lung damage (81, 82). NET formation and neutrophil elastase (NE) activity are elevated in TB patients with extensive tissue damage when compared to those with minor damage (83). NE degrades ECM and promotes fibrotic remodeling by inducing TGF-β production and myofibroblast differentiation (82, 84–86). While NETs can trap Mtb, they do not kill it; their DNA and proteases degrade the lung matrix, perpetuating inflammation. NET release, driven by type I interferon (IFN)-induced PAD4-dependent histone citrullination, is abundant in necrotic/caseating granulomas in humans and NHPs (76, 87, 88). Circulating NET markers, including myeloperoxidase–DNA, NE–DNA complexes, and H3Cit, are elevated in active TB patients with severe lung destruction and cavitary disease (83, 87–90).

Pulmonary fibrosis is another hallmark of pathology, driven by persistent inflammation and dysregulated wound healing during and after active infection. Fibrotic granulomas contribute to lung damage through excessive collagen deposition and tissue remodeling. Collagen levels vary among TB granulomas (91). In NHPs, two primary fibrotic granuloma morphologies have been described (92). Peripheral fibrosis, where a collagen cuff surrounds granulomas, restricting disease dissemination (92–95). In contrast, centrally fibrotic granulomas have a collagenous structure throughout the lesion. These are often associated with the sterilization of a granuloma and occur more frequently following treatment than prior to (92–94). Fibrosis is linked to TGF-β activity during infection and repair (93), and the neutralization of interleukin (IL)-10 in cynomolgus macaques was associated with increased granuloma fibrosis (96). In chronic lesions, alternatively activated (M2-like) macrophages secrete pro-fibrotic mediators, such as TGF-β1, IL-10, and PDGF, which activate fibroblasts and promote ECM deposition, encapsulating granulomas in fibrous tissue (97). While fibrosis can help contain bacilli, excessive collagen deposition stiffens the lung parenchyma, thickens the alveolar walls, and leads to restrictive lung physiology. Predictive modeling also suggests that macrophage-to-myofibroblast transition contributes to fibrosis (98). Thus, fibrotic granulomas simultaneously constrain bacterial spread and drive irreversible tissue remodeling, a defining feature of PTLD.

Th17 is a double-edged sword. In mice, IL-17 enhances the production of cytokines that induce the recruitment of IFNγ-producing cells (99), and mucosa vaccine-induced protection has been reported to be mediated through the induction of Th17 (100). Conversely, dysregulated IL-17 activity drives neutrophilic inflammation and tissue damage. Elevated IL-17A and Th17 activity in TB patients correlates with neutrophil-chemoattractant, MMP-1 induction, and lung pathology (101). Additionally, persistent IL-23/IL-17 signaling or repeated BCG exposure enhances IL-17 production, neutrophil influx, and worsens pathology; these events are reversed in IL-23p19-deficient or IL-17-blocked mice (102). IL-17-induced S100A8/A9 proteins further amplify neutrophil recruitment, with serum S100A8/A9 levels correlating with the severity of TB disease and inflammatory lung damage (103). Indoleamine-2,3-dioxygenase (IDO)-mediated tryptophan catabolism suppresses IL-23/IL-17 signaling, while loss of IDO activity is associated with exaggerated IL-17 responses and neutrophil-driven inflammation (104). Conversely, IDO inhibition has been reported to improve immune control and may reorganize pathological inflammation, suggesting complex, context-dependent roles for tryptophan metabolism in shaping Th17 biology (105).

The role of the host immune response in driving both the development and the progression of active TB suggests that the host itself can be a therapeutic target. Multiple biological and immunological pathways offer opportunities for intervention, particularly those involved in excessive inflammation and subsequent tissue injury. HDTs aim to modulate these pathways—either to enhance pathogen clearance, reduce immune-mediated damage, or both—when used in conjunction with conventional anti-TB drugs (14, 19, 106).

Over the past decade, several agents have been developed or repurposed for TB as HDTs, and several have been evaluated in pre-clinical studies and early-phase clinical trials. These agents are classified based on the biological processes they target, ranging from inflammatory signaling pathways to host metabolic functions. Because HDTs act on host mechanisms rather than directly on the pathogen, they are not susceptible to the emergence of drug resistance (13–19, 22, 62).

In TB management, HDTs may enhance bacterial killing while minimizing inflammatory tissue damage, acting either additively or synergistically with standard antimicrobials. Clinical evidence suggests that such adjunctive approaches—acting through novel mechanisms of action—could shorten treatment duration, improve outcomes in drug-resistant TB, and reduce transmission risk (13, 106). In post-TB contexts, these therapies hold particular promise for preventing or mitigating PTLD (62).

HDTs seek to rebalance pro- and anti-inflammatory immune activities: enhancing mycobacterial killing by immune cells, improving antimicrobial drug penetration into lesions, and curbing excessive inflammation to prevent irreversible damage (7, 13). By targeting fundamental and highly conserved host pathways, these therapies can address multiple disease stages, including controlling active infection, reducing pathological remodeling, limiting tissue injury, and potentially preserving long-term lung function (13, 14, 18). As interest grows, research into pharmacological strategies that modulate host–pathogen interactions in PTLD is expanding rapidly, with ongoing studies aiming to define the most effective interventions across different clinical scenarios.

Current HDTs under investigation or in clinical development span several mechanistic categories, which will be detailed in the following sections.

## INFLAMMATORY AND IMMUNE MODULATORS

### Metalloproteinase inhibitors

Necrotic granulomas are characterized by the accumulation of lipid-laden foamy macrophages within the granuloma foci. As these macrophages undergo necrosis, they release their lipid contents into the core, driving caseous necrosis and predisposing to cavitation (69, 70). MMPs, particularly MMP-1, MMP-3, and MMP-9, have been strongly implicated in ECM degradation and the progression from necrotic granulomas to cavitary lesions (71, 72, 75). In an experimental model system, adjunctive inhibition of MMP-9 has been shown to enhance the bactericidal effect of isoniazid (INH) and reduce lung bacterial load in murine models (106). Persistent MMP elevation in TB patients after completion of therapy may contribute to ongoing lung injury and progressive functional decline. Given the role of MMPs in other degenerative diseases, several targeted MMP inhibitors have been developed, some of which are now being evaluated as HDTs for TB.

Doxycycline, an FDA-approved antimicrobial, exhibits MMP inhibitory activity (22, 107). Doxycycline suppresses TB-induced MMP activation and limits mycobacterial growth in cell cultures and guinea pig models, where it reduced lung bacterial burden but did not alter the extent of granulomatous involvement (108). In a double-blind, randomized, and controlled phase II pilot trial—Doxy TB (NCT02774993), doxycycline was assessed as an adjunctive HDT for pulmonary TB. In this trial, doxycycline greatly reduced pulmonary cavity volume, lowered MMP levels in blood and sputum, and decreased elastase and type 1 collagenase activity (22). While earlier MMP inhibitors were limited by toxicity, newer generations are expected to offer improved tolerability.

Other relevant MMP inhibitors, such as Sb-3ct and marimastat, were found to work synergistically with INH and rifampicin to reduce bacterial burden in murine models and enhance lung delivery and maintenance of rifampicin and INH, while improving normal vascularization. In human lung tissue models, marimastat decreased early granuloma formation as well (109, 110). Despite these promising results, marimastat’s clinical development (BB-2516) was discontinued due to adverse effects. Interestingly, cipemastat, the inhibitor of MMP-1 and MMP-7, also demonstrated unfavorable effects in preclinical TB studies in the C3HeB/FeJ mouse model. Mtb H37Rv-infected C3HeB/FeJ mice treated with cipemastat showed increased lung pathology, cavitation, and mortality, just as rabbit studies confirmed its inability to prevent cavity formation (111), underscoring the relevance of this murine model for preclinical studies, investigating disease pathology, and the effects of HDTs on pathology and disease resolution. This notwithstanding, the next series of MMP inhibitors designed with greater specificity, improved safety profiles, and reduced off-target effects remains promising candidates for TB HDT development. These newer compounds may overcome the limitations that marred the usefulness of earlier drugs and represent a viable strategy to mitigate MMP-driven lung damage while enhancing the efficacy of conventional TB treatment.

### Tumor necrosis factor blockers

Tumor necrosis factor (TNF) is a critical cytokine involved in the formation and maintenance of granulomas during TB infection. However, excessive TNF levels can contribute to lung pathology, exacerbating tissue damage and inflammation. Consequently, therapies targeting TNF aim to mitigate these harmful effects (16, 112, 113).

One indirect strategy to control MMP activity and TB-associated inflammation is to modulate upstream regulators, such as TNF and the transcription factor NF-κB. TNF propels lung damage in TB by promoting granuloma maturation, cavitation, and the expression of MMPs (114–117). Importantly, elevated TNF levels correlate with poorer resolution of lung lesions during therapy. Furthermore, TNF contributes to pulmonary fibrosis and airflow obstruction (118). There are reports indicating that TNF inhibitors have improved clinical outcomes in advanced drug-susceptible TB (119). However, blocking TNF without concurrent multidrug anti-TB therapy risks impairing bacterial containment and worsening disease. Despite the potential of TNF antagonists as adjunctive treatments, the possibility of disease exacerbation when given without proper anti-TB drugs has limited advancement to large-scale clinical trials (120). Optimizing the timing and dosing of TNF blockers may help mitigate such adverse effects.

TNF-blockers include thalidomide and its analogs, which have demonstrated good tolerability in TB patients in placebo-controlled pilot studies, where they improved clinical outcomes such as body weight gain (121). Furthermore, etanercept, a soluble TNF receptor 2 fusion protein commonly used to treat rheumatoid arthritis, has also been evaluated as adjunctive therapy in TB (122). In C3HeB/FeJ mice, further emphasizing the relevance of this murine model in studying pathology and pathological changes after the administration of HDTs, etanercept administration, alongside standard TB drugs, reduced granuloma necrosis and accelerated lesion resolution compared to standard treatment alone in Mtb H37Rv-infected mice. Etanercept-treated mice exhibited earlier decreases in lung involvement without initial changes in bacterial load, suggesting an effect on early granuloma formation. The bacterial burden was significantly reduced during the continuation phase of treatment, likely reflecting enhanced killing of persister bacilli (119).

### Phosphodiesterase inhibitors

Phosphodiesterase (PDE) inhibitors also suppress TNF production, offering a potential strategy to modulate inflammation in TB. PDE inhibitors have shown promise when used adjunctively with standard anti-TB drugs, suggesting a role in combination therapy. When combined with INH, lung bacterial load reduces, lung pathology and fibrosis are ameliorated, and the size and number of granulomas decrease in infected rabbits and mice during both acute and chronic stages of infection (14).

Known and approved PDE inhibitors include sildenafil and cilostazol, both of which have been evaluated in murine TB models. Cilostazol showed more promising results and significantly reduced the mycobacterial burden in mice infected with Mtb. Combining cilostazol with sildenafil shortened the time required for lung sterilization and reduced pulmonary lesions. In C3HeB/FeJ mice infected with Mtb CDC1551, cilostazol and sildenafil caused a decrease in levels of TNF and IFN-γ and increased levels of IL-10 and IL-17 in lung homogenates of mice, relative to the levels in infected untreated mice, indicating that cilostazol and sildenafil had immunomodulatory effects on Th1 balance (123). Additional preclinical studies have demonstrated that cilostazol can reduce tissue damage, accelerate bacterial clearance, and enhance sterilization when administered in conjunction with standard TB therapy (123).

Other PDE inhibitors, when combined with standard therapy, have also shown promising results. Co-administration of roflumilast with INH resulted in reduced mycobacterial loads. Independently, CC-3052 or CC-11050, in combination with INH, significantly decreased lung pathology and bacterial counts in rabbits, characterized by fewer subpleural lesions, reduced necrosis, smaller granulomas, and less lung involvement. Granulomas in the combination group showed minimal necrosis, a central core of epithelioid macrophages, abundant lymphocytes, few acid-fast bacilli, and low fibrosis levels; however, CC-3052 alone increased lesion numbers and necrosis relative to untreated controls, suggesting the necessity of combination therapy for optimal benefit (124–126). Patients treated with CC-11050 as an adjunct to TB drugs showed moderate improvement in lung function (58, 127).

### IL-1 modulators

IL-1 is important for protective immunity against TB (128); however, exacerbated IL-1 levels lead to severe pathology characterized by extensive necrosis, neutrophil infiltration, and uncontrolled bacterial burden (129). Individuals with high IL-1-expressing genotypes, for example, rs1143627T, have been reported to be more prone to developing severe disease characterized by more cavitary lesions (130). Therefore, HDTs that modulate IL-1 levels warrant further investigation as a strategy to reduce TB-induced pathology; however, caution must be taken as IL-1 is also important for protective immunity against TB. In macaques and C3HeB/FeJ mice infected with Mtb Erdman, the FDA-approved IL-1 receptor antagonist anakinra, when administered alongside linezolid, reduced total lung inflammation, lowered the risk of reactivation, and mitigated linezolid-associated toxicity during infection, compared to linezolid alone (14, 131). Furthermore, combination therapy of TB drugs and anakinra reduced inflammatory markers and improved radiological readings in five out of seven HIV-negative individuals (132). However, more studies are needed to evaluate its application as a potential adjunctive therapy for preventing PTLD; furthermore, C3HeB/FeJ mice are an ideal model for these preclinical studies.

### Statins

Statins are known for lowering cholesterol in patients with cardiovascular disease; however, they were also found to exhibit broad immunomodulatory and anti-inflammatory effects, suggesting their potential use as HDTs for infectious diseases (14). In Mtb H37Rv-infected C3HeB/FeJ mice, statins lowered lung colony-forming units (CFUs) and decreased the percentage of lung surface area affected by inflammation, similar to findings in other mouse studies where statin treatment reduced Mtb burden by promoting autophagy, decreasing pulmonary pathology, and shortening the time to TB cure (14, 133). Population-based studies have also associated statin use with a reduced risk of developing TB. Other statins are in clinical trials and have shown promise for mitigating TB pathology (134, 135).

### Nonsteroidal anti-inflammatory drugs

Nonsteroidal anti-inflammatory drugs (NSAIDs), ibuprofen and aspirin, exert their effects mainly by inhibiting cyclooxygenase enzymes (COX-1 and COX-2) and are commonly used for the management of inflammation, fever, and pain. The C3HeB/FeJ model demonstrated that ibuprofen treatment effectively reduced both the number and size of lung lesions, lowered the mycobacterial burden, and improved survival rates in Mtb H37Rv-infected mice. Histopathological analysis revealed that ibuprofen-treated animals exhibited increased intra-alveolar neutrophil infiltration and thickened alveolar walls, in contrast to control mice, which showed large central areas of caseous and liquefactive necrosis (136).

Intriguingly, ibuprofen alone did not alter bacterial burden in Mtb H37Rv-infected BALB/c mice; however, it caused a reduction in granulomatous lesions in the Mtb H37Rv-infected C3HeB/FeJ model, reflecting its anti-inflammatory effects and affirming the C3HeB/FeJ mouse model as well-suited for probing the effects of HDTs on TB immunopathology, further. Interestingly, low-dose aspirin, either alone or in combination with other TB drugs, has been shown to improve survival, reduce lung pathology, and decrease bacterial load in the C3HeB/FeJ model during chronic Mtb infection (14, 136–138). Both ibuprofen and aspirin enhanced the antimycobacterial activity of pyrazinamide in Mtb H37Rv-infected mice. However, combining aspirin with INH, in BALB/c mice, increased bacterial counts in these mice compared to INH alone (137, 139).

Despite some of the promising findings with these NSAIDs, adverse effects have been reported as discussed, highlighting the need for additional preclinical—in relevant models—and clinical studies to determine their true impact on both active and latent TB.

### Vitamin D

In its biologically active form, 1,25-dihydroxyvitamin D (vitamin D) regulates mucosal immunity and inflammation by binding to the vitamin D receptor and triggering the production of the antimicrobial peptide cathelicidin, which facilitates autophagosome-lysosome fusion and maturation. By directly inhibiting bacterial growth and enhancing innate immune responses, vitamin D plays an important role in controlling Mtb infection (19).

In macrophages, vitamin D induces a phenotypic shift from the pro-inflammatory M1 state to the wound-healing reparative M2 phenotype, promoting the secretion of hydrogen peroxide, which aids in eliminating Mtb. Proinflammatory cytokines and chemokines are downregulated via suppression of NF-κB signaling, while promoting anti-inflammatory cytokines in pulmonary TB. It also suppresses Th1 and cytotoxic T cell responses, promoting the differentiation of T regulatory cells, and thereby reducing IFN-γ and IL-17 production (15, 140, 141).

Supplementation with cholecalciferol (vitamin D₃) may enhance standard anti-TB therapy by accelerating sputum culture conversion and improving lesion healing, as demonstrated in some studies (19, 142). High-dose vitamin D administration, adjunctive to standard anti-TB drugs, improved clinical and radiological outcomes in individuals with TB. Despite these promising findings, while some clinical trials have shown positive results, others have not shown accelerated sputum culture conversion following vitamin D supplementation. In fact, some reported no significant effects on culture conversion or TB relapse rates. Notably, however, genotype effects were found to significantly influence the effect of vitamin D in participants, hastening sputum culture conversion and indicating that vitamin D supplementation could still be beneficial as an adjunctive therapy (15, 143–145).

That said, vitamin D promotes the production of reactive oxygen and nitrogen species and antimicrobial peptides while depriving Mtb of iron, limiting lipid droplet accumulation in infected macrophages. Moreover, vitamin D upregulates autophagy, facilitating autophagosome initiation and lysosomal fusion to restrict Mtb replication (15).

The effects of vitamin D have been demonstrated in the C3HeB/FeJ model, which we propose as a model for studying the efficacy of HDTs. In this model, dietary cholecalciferol decreased pulmonary immunopathology and altered immune cell profiles in lung granulomas of mice during chronic infection (14). Although Mtb bacterial burden did not differ between mice on a VitD_3_-replete diet and mice on a deficient diet, the pro-inflammatory response in mice that received VitD_3_ was significantly reduced relative to controls. This was followed by a concomitant reduction in pulmonary immunopathology in mice on a VitD_3_-replete diet. Additionally, VitD_3_ supplementation limited accumulation of IFNγ- and TNF-producing CD4^+^ (i.e., T_H_1) cells (146). When mice were infected with Mtb Erdman and given VitD_3_, more CD8^+^ T cells and CD11b^+^Gr1^+^ neutrophils were found in their lungs. Moreover, aggregates of lymphocytes in the inflammatory zones were significantly smaller in the lungs of VitD_3_-treated mice compared to those of control mice. Interestingly, however, there was no decrease in bacterial burden at 6 weeks in the VitD_3_-treated mice, unlike in control untreated mice, and this was significant between the two groups of mice (147). Given that these studies were carried out in C57BL/6 mice, which do not accurately recapitulate human disease and pathology, it would be important to repeat this study in the more representative C3HeB/FeJ model that better predicts the course of disease and immunopathology observed in humans.

### Omega-3 long-chain polyunsaturated fatty acids

Omega-3 long-chain polyunsaturated fatty acids (n-3 LCPUFAs) are essential for regulating inflammatory responses and are abundant in oily fish and as dietary supplements (19). The C3HeB/FeJ murine model was instrumental in demonstrating that incorporating n-3 LCPUFAs into treatment regimens can lower Mtb burden, reduce systemic and pulmonary inflammation, and improve body weight in Mtb H37Rv-infected C3HeB/FeJ mice with adequate baseline n-3 LCPUFA status. Importantly, this model demonstrated that n-3 LCPUFAs alone, but not in combination with iron supplementation, were capable of producing the desired outcomes (148–150).

The C3HeB/FeJ model highlighted key factors that must be considered when evaluating the administration of any HDT to achieve desired results. It provided important insights into variables that must be carefully considered in the evaluation of HDTs for TB. These include the timing of HDT initiation, the dose administered, and whether the genetic background of the experimental animal model adequately recapitulates the pathological hallmarks of human pulmonary TB. Notably, this model revealed results divergent from those observed in other murine and non-murine systems, underscoring the need for critical appraisal of model-specific influences when interpreting preclinical data assessing the efficacy of TB HDTs (150).

## SIGNALING AND KINASE PATHWAY INHIBITORS

### Kinase inhibitors

The tyrosine kinase inhibitor, imatinib, approved by the FDA for treating leukemia, has shown potential against mycobacterial infections as well. In Mtb-infected mice, imatinib reduced the mycobacterial burden, lowered lysosomal pH, thereby restricting Mtb growth in humans, and in combination with rifampicin, synergistically suppressed intracellular Mtb survival in infected THP-1 macrophages (151, 152). In a mouse model infected with Mtb Erdman, imatinib reduced the bacterial load by up to about 60-fold. In fact, bacteria were below the limit of detection when plated on agar for more than 50% of the mice. Furthermore, more than 70% of mice showed limits below 10^5^ CFUs, compared to controls that showed up to 80% more bacteria. Furthermore, additional confirmatory experiments showed a 185-fold reduction in bacterial load in mice treated with imatinib compared with controls, with a significant number of imatinib-treated mice again showing CFU counts below the limit of detection. Together, these data showed that imatinib was an effective HDT against Mtb in severely infected mice (152).

Promising *in vitro* and *in vivo* results for imatinib led to it being moved into human trials, IMPACT-TB (NCT03891901), to assess safety, pharmacokinetics, and effects of imatinib on myelopoiesis in adults. The trial involved administering imatinib with and without INH and rifabutin, with the aim of defining the dose to be studied in a subsequent Phase IIB treatment trial evaluating imatinib as adjunctive therapy with a TB drug regimen comprising rifabutin, pyrazinamide, INH, and ethambutol for susceptible TB. Although the trial was closed in 2022, its findings are yet to be shared with the wider scientific community and the general populace (153).

Gefitinib and ibrutinib are other tyrosine kinase inhibitors that have demonstrated anti-TB activity and reduced lung CFU counts, including lymph node and spleen coinfection murine TB models, while limiting intracellular Mtb replication in macrophages. Both gefitinib and ibrutinib have been shown to be associated with the induction of autophagy (14). In C57BL/6 mice treated with ibrutinib after Mtb H37Rv infection, there was no significant reduction in bacterial load in the lungs of mice relative to control; however, there was a significant decrease in the mediastinal lymph node and spleen (154). Given that C57BL/6 mice do not completely recapitulate the full spectrum of disease and its manifestations, further studies in the C3HeB/FeJ model, which more closely recapitulates human disease and response to drugs or compounds, are warranted.

### Vascular endothelial growth factor inhibitors

Uncontrolled activation of vascular endothelial growth factor (VEGF)-A-driven processes leads to the generation of dysfunctional blood vessels, which contribute to hypoxia, impair the penetration of anti-tubercular drugs, and limit immune cell recruitment (14). VEGF production is stimulated by Mtb through virulence factors, including the ESX-1 secretion system and trehalose dimycolate, suggesting that Mtb deliberately exploits angiogenic pathways to aid dissemination (14).

Mtb infection drives extensive remodeling of host vasculature, promoting the formation of new, but structurally and morphologically abnormal, blood vessels around granulomas. Morphologically, granulomas parallel solid tumors, with both exhibiting hypoxic microenvironments and fibrosis. These granuloma-associated vessels have highly variable spatial densities, and the aberrant vascularization benefits the pathogen by supporting its growth and facilitating dissemination. This aberrant vasculature severely restricts the penetration of small molecules, with tracer compounds accumulating mainly in peripheral lesion regions (14, 155).

The VEGF inhibitor bevacizumab targets all VEGF-A isoforms and is FDA approved for treating multiple cancers. In a rabbit model of TB, bevacizumab promoted vascular normalization within granulomas, reducing hypoxia and enhancing penetration of small molecules, including fluorescent dyes, into granulomatous tissue, highlighting its potential as a promising adjunctive therapy, though it was not found to significantly reduce lesion volume, inflammation, or mycobacterial burden (155).

Again, highlighting the relevance of the C3HeB/FeJ mouse model for probing the efficacy of HDTs, their impact on pathology, bacterial clearance, and disease resolution, blocking VEGFR1 and VEGFR2 signaling with SU5416 in Mtb H37Rv-infected C3HeB/FeJ mice resulted in granulomas that were smaller and less dense, with reduced obstruction of alveoli and blood vessels. In contrast, granulomas in C3HeB/FeJ mice without the therapeutic intervention were caseating, with their hypoxic regions staining positively for VEGF-A. Critically, though, it was observed in this mouse model that treatment with SU5416, paradoxically, was associated with a significant increase in mycobacterial burden compared to vehicle-treated controls, showing how this murine model can provide a comprehensive picture of the full scope, effect, and impact of HDTs on infection and pathology (156).

### Histone deacetylase modulators: resveratrol and SRT1720

Resveratrol reverses the suppression of sirtuin 1, which protects cells from apoptosis, and exerts anti-inflammatory effects by inhibiting pathways such as NF-κB and the production of IL-6 and TNF (157–160). Resveratrol was also shown to reduce bacterial burden, improve lung pathology, and synergize with anti-TB therapy to enhance bacterial clearance (14). SRT1720, like its natural counterpart resveratrol, alleviated lung pathology and worked well with TB drugs to enhance their effect and reduce chronic inflammation (14, 161). Treatment with resveratrol or SRT1720 led to similar improvements in pathology (161). In macrophages and C57BL/6 mice, sirtuin 1 activation suppressed intracellular Mtb growth and bacterial load, respectively, while stimulating autophagy, when Mtb H37Rv, or Erdman, was studied (161). Conversely, inhibition of sirtuin 2 with AGK2 reduced Mtb H37Rv bacterial growth and load in *ex vivo* peritoneal macrophages from C57BL/6 mice and in BALB/c mice, respectively (162). The opposite effect observed with these compounds warrants further investigation in relevant animal models, such as the C3HeB/FeJ murine model, which more closely mimics human pathology in response to TB infection and disease caused by different Mtb strains, as we and others have reported previously (163–166).

## METABOLIC MODULATORS

### Rapamycin and its analogs

Using C3HeB/FeJ mice as an experimental model to investigate HDTs, the potential role of rapamycin, an mTOR inhibitor and autophagy inducer, as an adjunctive candidate was assessed in Mtb Erdman-infected mice. The study found that co-administering rapamycin and moxifloxacin reduced infection-driven lung inflammation and the number and size of caseating necrotic granulomas, without significantly affecting the lung bacterial burden (167). Rapamycin given in the chronic phase of disease, which most closely resembles the clinical situation in which TB therapy would be augmented with an HDT, produced results consistent with a trend toward decreased CFUs in rapamycin-treated mice. Furthermore, histopathological examination of the lung tissue revealed significantly reduced inflammation and immunopathology in rapamycin-treated mice. These mice exhibited fewer and smaller caseating necrotic granulomas that were confined to only sparse areas of the lung. Furthermore, in assessing the role of rapamycin as adjunctive therapy administered alongside standard TB drugs, coadministration of rapamycin and moxifloxacin was evaluated, and again, histopathological evaluation revealed a significant reduction in lung inflammation and immunopathology in animals that received combination treatment of rapamycin and moxifloxacin compared to controls that received moxifloxacin along with the control diet (167).

In another study, mice cotreated with clofazimine and rapamycin were cleared of both multiple and extensively drug-resistant clinical isolates of Mtb, and this was attributed to the induction of robust T-cell memory and polyfunctional T-central memory responses (168). Notably, patients treated with everolimus, an mTOR inhibitor, or CC-11050 as an adjunct to TB drugs showed a moderate improvement in lung function, despite treatment not affecting sputum culture conversion status *per se* (58, 127).

### Metformin

Metformin, first-line therapy for type 2 diabetes, has emerged as a promising host-directed adjunct therapy in TB. Across epidemiological studies, animal models, and early clinical trials, metformin has been associated with reduced risk of developing TB disease, attenuation of immunopathological responses, and fewer cavitary lesions.

In a study comparing TB patients with diabetes (TB-DM) who received metformin with those not on the drug, metformin treatment was associated with fewer pulmonary cavities and reduced mortality (169). Supporting evidence from a murine model (C57BL/6) showed that metformin reduced chronic inflammatory lesions and ameliorated tissue pathology. Metformin reduced intracellular bacterial growth in an AMPK-dependent manner (169). The protective effect was associated with increased ROS production, enhanced phagosome acidification, and higher frequencies of Mtb-specific IFNγ-secreting CD8^+^ T cells, with a trend toward increased CD4^+^ T cells (169). Metformin has also been reported to enhance anti-mycobacterial responses by reprogramming the metabolic and transcriptional circuits of CD8 T cells and expanding memory-like CD8+CXCR3 T cells in mice and humans (170).

In guinea pigs with chronic Mtb infection, metformin treatment lowered lung CFUs and led to well-organized granulomas, with distinct granuloma architecture and increased lymphocyte infiltration, while showing reduced inflammation. However, metformin caused a late, but not early, reduction in bacterial load (14).

Additional clinical evidence supports an immunomodulatory role of metformin. Systemic monocyte inflammatory markers, including sCD14, sCD163, and CRP levels, were significantly elevated in TB-DM individuals with cavitary disease and showed a positive correlation with bacterial burden (171). Metformin treatment reduced systemic levels of sCD14, sCD163, and CRP in diabetic patients with TB compared to those not receiving metformin (171). Similarly, MMPs, particularly MMP-1, MMP-2, MMP-3, and MMP-12, were significantly elevated in diabetic TB patients with cavitary disease but were markedly reduced with metformin treatment (172).

In a randomized clinical trial, the addition of metformin to standard anti-TB therapy significantly reduced cavitary lesions on chest X-rays and lowered inflammatory markers (56). Currently, metformin is being evaluated as an adjunctive therapy for rifampicin-resistant TB with efficacy endpoints, including lung function recovery and eradication of Mtb infection (173).

## CHALLENGES AND CONCERNS IN THE DEVELOPMENT OF HDTs FOR TB

While the concept of modulating the host response to improve TB outcomes is compelling, accumulating evidence reminds us that immune interventions can be double-edged, with the potential to worsen disease if applied without guidance and caution. There is some evidence that their translation is far from straightforward, and that their benefits are neither universal nor guaranteed in every case. As adjuncts to conventional TB treatment, targeting the host rather than the pathogen, critical questions about their reliability are being addressed across the board. Recent preclinical and clinical findings highlight important uncertainties regarding safety, timing, and patient-specific effectiveness. Persistent gaps in mechanistic understanding and inconsistent clinical performance underscore the need for a more critical evaluation.

For instance, there have been concerns about the potential for harm by driving dangerous immunopathological responses or increasing bacterial growth. Some HDTs have been shown to dysregulate host defenses and worsen infection. Immunomodulation can worsen disease if the mechanism or timing is wrong, and animal models have revealed some of these paradoxical effects ahead of clinical trials.

This phenomenon has been shown with phosphodiesterase inhibitors (PDE-Is). Multiple studies have shown the benefit of administering certain PDE-Is, such as those targeting PDE3 and PDE5 (123, 126, 174, 175), whereas PDE4-Is were not favorable and, in fact, were found to be harmful, both alone and in combination with standard TB drugs. Treatment with PDE4-Is, rolipram and cilomilast, accelerated the time to death for Mtb-infected BALB/c mice. Administering rolipram increased bacterial burden, did not improve the time to bacterial clearance in the lung, and led to higher relapse rates compared to other treatment groups (176). Indeed, only roflumilast, the single PDE4-I approved by the FDA for treating COPD, had been in use; however, even with this drug, its use was limited due to psychiatric and gastrointestinal adverse events observed, consequently resulting in the discontinued use of other PDE4-Is, such as rolipram and cilomilast, in human studies (176).

PDE4s, known to be anti-inflammatory in their effect, encode 4 genes, PDE4A-D, that also encode altogether more than 15 different isozymes, differentially expressed in relation to the cell and tissue environment (177). Therefore, the specific PDE4 variants inhibited by rolipram, cilomilast, and CC-3052 may vary. CC-3052 is a potent inhibitor of TNFα (178), and inhibition of TNF has been strongly linked with reactivation of latent TB, underscoring the need for careful consideration before administering PDE4-Is to patients with active or latent TB (15).

Similarly, there have been concerns about administering the autophagy inducer, rapamycin (sirolimus), which, though it reduces bacterial replication in macrophages, also leads to significant immune suppression and is metabolized by the cytochrome P450 enzyme CYP3A4. This enzyme is strongly induced by rifampicin, warranting caution for its use in combination with the anti-TB drug, as rifampicin is part of the standard TB treatment regimen and would be difficult to avoid during adjunctive therapy (15, 179). Furthermore, there are concerns about reactivation of latent TB infection upon rapamycin therapy (180, 181), and these concerns have not only been expressed for rapamycin but also for other TB HDT candidates that have been administered in immunosuppressed patients, such as individuals with cancer, undergoing chemotherapy (182, 183).

Other translational issues raised have been related to dosing and timing uncertainties and animal model limitations leading to false-positive or negative results when moved into human studies and clinical translation. The lack of relevant and appropriate clinical biomarkers to categorize patients to make a right assessment of which patients are most likely to benefit from specific adjunctive therapies is a further limitation.

In *in vivo* studies, it was observed that the administration of high-dose vitamin D (15 mg or 600,000 IU for 2 doses) alongside standard TB medication improved patients’ clinical outcomes and radiological results, suppressed proinflammatory cytokine production, and sped up the resolution of inflammatory responses during treatment (184, 185). However, in another study with a different dose, which was also referred to as high dose by the team of investigators, the same beneficial effect was not observed. Vitamin D supplementation administered at 1.25 mg (50,000 IU) alongside TB drugs did not alter culture conversion rates in pulmonary TB patients significantly (186), and this phenomenon was observed in other studies (143, 145), highlighting the potential impact of dosing differences in influencing the outcome of adjunct therapy with HDTs. Granted, other factors, some of which have been reported, such as genotypic differences (143), could have driven the differences observed. Nonetheless, the role proper dosing plays in determining the outcome of therapy cannot be overlooked. The impact dosing has on the outcome of therapy was confirmed in animal studies where doxycycline treatment significantly reduced the lung bacterial burden in guinea pigs in a dose-dependent manner (108).

The dose-dependent effect was also seen in the administration of metformin, where many studies showed significantly reduced incidence of active TB in diabetic patients, with a superior protective effect of high-dose compared to low-dose metformin (187–189).

The timing of HDT administration is also critical. In the two different vitamin D studies that showed contrasting results when given adjunctively with standard TB therapy, the study showing favorable outcomes in patients administered two doses of vitamin D (15 mg or 600,000 IU) a month apart, while the study reporting unfavorable results administered vitamin D at a lower dose (1.25 mg or 50,000 IU) three times weekly for 8 weeks and another 50,000 IU every other week for 8 weeks (185, 186). In a study comparing statin adjunctive therapy with standard TB treatment alone for up to 4.5 months, it was observed that following treatment with standard TB drugs alone, BALB/c mice that received treatment for 1.5, 2.5, and 4.5 months showed a reduction in lung CFU of 3, 4.7, and 5.8 log_10,_ respectively, relative to untreated mice that showed between 5.7 and 5.9 log_10_ CFU, up to 4.5 months post-treatment. In this study, when simvastatin was administered adjunctively, with standard treatment over the time course, CFU reduced by an additional 1.4 log_10_, 0.64 log_10_, and 0.5 log_10_, respectively (133).

Taken together, these studies emphasize the complex dynamics and critical role that dosage and timing of HDT administration play in determining the trajectory of treatment outcomes during adjunctive therapy.

Beyond dosage and timing, another concern has been the lack of appropriate clinical biomarkers. This remains a challenge for HDT translation. Biomarkers enable rational decisions to be made about selecting appropriate therapy for patients and adjusting drug doses (190). It has been proposed that biomarkers will become an integral part of future therapy regimens and thus must be prioritized in infectious diseases drug research and development efforts, be they host or pathogen directed (179). As markers that provide information on the stage of infection, enable monitoring of treatment outcomes, furnish information on organs and specific areas affected, and the type of inflammation, while allowing patient selection or stratification for specific HDTs, biomarker discovery will be critical to the success of HDT clinical translation and epidemiological impact (179, 191). For instance, an HDT approach to treat people with latent TB, as adjunct therapy in combination with antimicrobial therapy, either to improve efficacy or to shorten the window of antimicrobial treatment while reducing risk of progression to active disease, has been proposed; however, its translation would have to be informed by peculiarities of the patient population and would require biomarkers to make this strategy possible to implement (191, 192).

Indeed, most agents re-purposed as HDTs have some documented safety profiles, as many have been studied in the field of cancer research. However, within the context of TB, their profiles have yet to be generated. It is known that malnutrition, co-infection with HIV, diabetes, and inflammation impact drug metabolism, and all these factors are strongly associated with TB disease and its progression; therefore, these cannot be overlooked in the clinical translation of HDTs. Co-administration of HDTs and TB drugs must be evaluated for contraindications and toxicity, and drug-drug interactions with antiretroviral therapy, as an example, must be determined, given that TB and HIV are strongly associated (15, 54, 179, 190, 193). Furthermore, rapamycin activates macrophages to boost activity against Mtb; however, its effect is impeded in diabetic patients (194).

Other concerns have centered on contradictory study results and limited or underpowered studies and clinical trials.

When the effect of statins as adjunctive therapy for TB was investigated in a retrospective cohort study, no significant association between statin use and the risk of developing TB in diabetic patients was observed, and statin administration was weakly associated with the risk of TB in elderly diabetic patients (195, 196). Contrary to these findings, individuals given statins had a significantly lower risk of developing TB than controls who were not administered statins, although the observed protective effect was dampened by diabetes (197). Another group found similar outcomes and reported that this protective effect was dose-dependent (198).

Again, in studies reporting on the use of metformin, mixed clinical results, with some trials and cohorts showing improved outcomes or reduced inflammation and others reporting modest to no effect, have raised concerns about consistency, optimal dosing, and which patient groups truly benefit. In one trial, METRIF, administering metformin adjunctively with standard TB therapy, did not shorten the time to sputum culture conversion; however, it reduced inflammation and subsequently lung tissue damage, evidenced by faster resolution on chest radiographs along with a reduction in inflammatory markers (56), while in another study, metformin was found to have a slight protective effect on sputum conversion after 2 months of standard TB therapy in patients with diabetes, although one limitation with this study was its small sample size (199). It was reported that coadministration of metformin with INH had a more potent effect on cytokine and chemokine modulation than INH alone, significantly restricting bacterial growth (20). In two different cohorts where metformin was given adjunctively, the efficacy of standard TB drugs was improved, leading to better outcomes (169).

Together, these contradictions warrant the need for well-designed studies, with appropriate models and clinically relevant endpoints, to inform clinical translation of HDTs.

## FUTURE PERSPECTIVES—HARNESSING C3HEB/FEJ MICE AS A PRECLINICAL MODEL FOR PTLD

To fully understand the mechanisms leading to PTLD and cost-effective ways to test therapeutic interventions, small animal models are necessary. Several of the HDTs described in the previous section employed the C3HeB/FeJ mice, thereby supporting the development of C3HeB/FeJ mice as a pre-clinical model for PTLD. Most mouse strains do not develop the hallmark lung cavitation and caseating granulomas seen in humans. Instead, murine granulomas are generally non-hypoxic, lack central necrosis, and are composed mainly of loosely organized macrophages, T lymphocytes, and sparse fibrous tissue. An exception, though, is the highly susceptible C3HeB/FeJ strain, which develops organized, hypoxic lung lesions with central liquefactive necrosis and extracellular mycobacteria following Mtb infection (200). These lesions are surrounded by a fibrous capsule and bear a strong resemblance to human TB pathology, making this model particularly valuable for testing anti-TB agents—especially those targeting bacilli that persist within necrotic lesions for studying post-TB disease sequelae (14, 200). Following TB infection, multiple lesion types with extremely different microenvironments closely resembling those seen in humans have been observed in C3HeB/FeJ mice, supporting the relevance of the C3HeB/FeJ mouse model for studying PTLD (163). This study showed that the lesions developed in the C3HeB/FeJ mice recapitulated the pathophysiological features of human TB lesions, including the development of caseous necrosis followed by collagen deposition and the development of hypoxic lesions. Some of these findings were reported in other studies as well, which showed that C3HeB/FeJ mice form cavities and display lesion heterogeneity and varied lung pathologies, closely mirroring human TB as visualized by CT imaging (200–204). A recent paper reported fibrogenesis, a key feature of pathological remodeling that is seen in human disease, in Mtb-infected C3HeB/FeJ mice (205). Together, these reaffirm leveraging the C3HeB/FeJ model as a relevant tool to study the effect of HDTs on disease pathology.

C3HeB/FeJ mice have also been used to study Mtb strain heterogeneity. We previously showed that high (Mtb-HT) and low (Mtb-LT) transmission strains induce distinct lung pathological responses in C3HeB/FeJ mice. Mtb-HT induced caseating necrotic granulomas with potential to form cavitary lesions, while Mtb-LT induced diffuse inflammatory lesions with neutrophilic infiltration (166, 206). This strain of mouse models heterogeneity in pulmonary deficits observed in people treated for TB (207), furthering its utility as a preclinical model for developing HDTs.

Significant improvements and innovation in technologies to assess lung function in mouse models can recapitulate the key pathological features of parenchymal disease via changes in compliance, functional residual capacity, total lung capacity, and vital capacity of the respiratory system (208). Many factors may influence the development of the heterogeneous lung damage with chronic inflammation and tissue destruction, including Mtb strain differences and the ensuing host-pathogen interactions, co-morbidities such as diabetes and malnutrition, environmental factors such as smoking and air pollution, and host genetics. Future studies should incorporate these variables and refine tools to study cellular and molecular pathways leading to the heterogeneity of airway and parenchymal lung damage, as well as subsequent consequences to lung function in the C3HeB/FeJ mice, for furthering its use as a preclinical model for evaluating HDTs targeted at preserving lung function and preventing PTLD.
